# Observations of the foraging behavior and activity patterns of the Korean wood mouse, *Apodemus peninsulae*, in China, using infra-red cameras

**DOI:** 10.3897/zookeys.992.57028

**Published:** 2020-11-12

**Authors:** Dianwei Li, Jingwei Hao, Xu Yao, Yang Liu, Ting Peng, Zhimin Jin, Fanxing Meng

**Affiliations:** 1 College of Life Sciences and Technology, Mudanjiang Normal University, No. 191 Wenhua Road, Mudanjiang, Heilongjiang 157011, China Mudanjiang Normal University Mudanjiang China; 2 Heilongjiang Academy of Forestry, No. 134 Haping Road, Harbin, Heilongjiang 150081, China Heilongjiang Academy of Forestry Harbin China

**Keywords:** Activity rhythm, feeding behavior, Glires, infra-red camera

## Abstract

*Apodemus peninsulae*, a dominant rodent species in temperature forests of northeastern China, is a model animal to explore the ecological functions of reciprocal coevolution of animals and plants. From August to October 2016, 24 infra-red cameras were installed to study the feeding behavior and activity patterns of *A. peninsulae* in its natural environment. By analyzing 5618 video records, we found that feeding behavior, followed by motor and sentinel behaviors, was their main activity. In the behavior spectra, motor behavior (creep, walk, and skip), feeding behavior (forage, feeding, transport, hoarding, and clean), and sentinel behavior (alert, flee, banishment, and coexistence) accounted for 57.96%, 40.36%, and 1.68% of their behavior, respectively. The peak of feeding behavior occurred between 18:00 and 23:00, and feeding behavior frequency, duration, and activity rhythms differ among August to October. Furthermore, activity was the greatest after sunset and before sunrise, indicating a nocturnal lifestyle; however, from August to October, the start time of the activity was earlier, and the end time was later than usual. On average, mice spent 21.6 ± 11.6 times/night feeding, with a duration of 63.58 ± 98.36 s; while they spent less time in foraging, 39.05 ± 51.63 s. We found a significant difference in feeding and foraging frequency, with mice spending on average 10.84 ± 9.85 times/night and 9.23 ± 11.17 times/night, respectively. Our results show that feeding and foraging behavior is also influenced by light intensity, suggesting a preference for crepuscular periods of the day. Infra-red cameras are very useful in detecting activity patterns of animals that are not easily observable; these cameras are able to capture a large amount of valuable information for research into ecological functions.

## Introduction

Through adaptive evolution, animals respond to environmental factors, as well as their physiology, in order to adapt to their environment (Lehner 1996; Halles and Stenseth 2000; Pearson and Theimer 2004; Seri et al. 2018; Tang et al. 2020). They do this in a range of ways, including adjusting their behavior in response to environmental changes, such a daily circadian rhythm, temperature, competition, predation avoidance, and resource availability, as well as their own physiology (Flannigan and Stookey 2002; Li et al. 2019; Tang et al. 2020). As a result, they form specific behavior patterns and activity rhythms. Therefore, it is important to understand how animals adapt and adjust their behavior to their environment and to assess the mechanisms that drive these patterns.

Foraging activity of animals involves a wide spectrum of behaviors used in the quest to find food. Foraging and feeding behaviors are often linked together, and animals will use a combination of strategies. Foraging and feeding behaviors include finding, obtaining, processing, ingesting, and hoarding, following specific pattern specific to the animal. Depending on the need, feeding can occur at the site where food is found or can be moved and consumed somewhere else. The decision to do so is dependent on external pressures such as predation, the time available for foraging, and environmental factors such as temperature (Halles and Stenseth 2000; Pearson and Theimer 2004; Seri et al. 2018). In learning the factors that affect the feeding and foraging behavior spectra of animals, we hope to gain further insight into adaptive capability, activity intensity, which will give us a clearer understanding on adaptive strategies of animals as well as niche dimensions in specific communities (Lehner 1996; Halles and Stenseth 2000; Pearson and Theimer 2004; Seri et al. 2018; Tang et al. 2020).

Infra-red (IR) cameras have been found to be highly beneficial to ecological studies of cryptic animals, those that are active at night when it is difficult to see them, or those that occur in hard to reach locations. Moreover, IR cameras have many benefits, such as being non-invasive, capacity for long-term monitoring with 24 hour or longer monitoring (Karanth 1995; Rowcliffe et al. 2008; O’Connell et al. 2020), low cost, low environmental interference, resistance to environmental changes, and ability to record greatly hidden species and complex terrain (Karanth 1995; Rowcliffe et al. 2008; Mondol et al. 2009; O’Connell et al. 2010; Zhang et al. 2013; Claridge et al. 2019). In addition, IR cameras can capture a large amount of information in the natural state (O’Connell et al. 2010), including population structure, species diversity, and some information on spatial distribution (Mondol et al. 2009; Zhang et al. 2013; Claridge et al. 2019). As a result, IR cameras have been widely used in studies involving field monitoring, quantity estimation, habitat characteristics, individual recognition, behavior patterns, and activity rhythm (Mondol et al. 2009; Zhang et al. 2013; Claridge et al. 2019). For example, in studies involving mammals in the superorder Glires, IR cameras have been widely used to assess species diversity and estimate population density (Zhang et al. 2013). However, in smaller-bodied species of Glires, such information is lacking. Much of the research has been conducted on larger species of Glires: Sino-Mongolia beaver *Caster fiber birulai*, Asiatic brush-tailed porcupine *Atherurus macrourus* and red-bellied squirrel *Callosciurus erythralus* (Liu et al. 2015; Wen et al. 2016; Wang et al. 2019; Tang et al. 2020). Thus, more research is needed using IR cameras to study the behavior of smaller-bodies species of Glires.

The Korean wood mouse, *Apodemus peninsulae*, is a dominant species of the Glires community in temperate forests in northeastern China, inhabiting a variety of habitats such as forests, shrubs, glades, grasslands, and farmlands at the forest margins. This species feeds on and stores a variety of seeds and fruits from plants such as *Quercus mongolican*, *Pinus koraiensis*, and *Corylus mandshurica* (Lu and Zhang 2005; Park et al. 2014; Liu et al. 2015; Li et al. 2018). As a keystone species in the forest ecosystem, *A. peninsulae* is not only a primary consumer and seed disperser, but also an important food resource for carnivores. Research on *A. peninsulae* has mainly focused on population dynamics, food storage, nest activity, body temperature, and distribution (Lu and Zhang 2005; Park et al. 2014; Liu et al. 2015; Li et al. 2018; Masaki et al. 2005). However, there were no reports on feeding behavior and activity patterns in *A. peninsulae* under natural conditions. However, Lu and Zhang (2005) did report on the activity rhythm and seed hoarding of this species under laboratory and artificial fence conditions. Due to the limitation of space and human interference, these studies could not fully reflect the natural activity rules of Gires. Our study used IR cameras to monitor the feeding behavior of *A. peninsulae* in its natural environment. Our results will provide a new perspective on the various application of IR cameras as a non-invasive tool for monitoring and studying the ecology of animal behavior in natural settings.

## Site and methods

### Study area and research site selection

The study was conducted from June 2015 to October 2016. The research site was in a forested area of Hengdaohezi town, Hailin City (44°44'N–44°55'N, 129°6'E–129°15'E, elevation 460–600 m), at the northern end of the Changbai Mountains in northeastern China, the east vein of the main ridge of Zhangguangcai Mountain. The mountain runs northwest-southeast. The climate is temperate continental monsoon, with four distinct seasons and a hot rainy season. The maximum temperature is 37 °C, the minimum temperature is –44.1 °C, and the annual average temperature is 2.3–3.7 °C. About 100–160 days in the year are frost free. The first frost is in late September, and the last frost is in late April to early May. Precipitation is concentrated in June to September and varies between 400 mm and 800 mm. The forests in this area are dominated by secondary vegetation. There is a high abundance and diversity of Glires, largely dominated by combinations of various species such as *A. peninsulae*, *A. agrarius*, and *Clethrionomys rufocanus*.

In the theropencedrymion, three alternative plots with less human interference were selected for research. For each plot, we first used the rat traps method to investigate the presence of small Glires in the area. The plot with the largest capture rate of *A. peninsulae* was selected as the research site. The chosen site was 100 m × 150 m and at an altitude of 533–552 m.

### Infra-red camera settings

In the study plot, four sample strips with an interval of 20 m were set, and six IR cameras (Ltl Acorn, LTL-6310MC) were installed in each sample strip. The IR camera interval was 20 m, with 24 cameras in total. The IR cameras were set to the photo + video mode. Each camera was set to take three shots after triggering and then to automatically record for 15 s after every 30 s interval. Cameras automatically recorded the date, time, environmental temperature, and other information. Cameras were fixed on tree trunks, or other fixed objects, about 30 cm above the ground, and baits (marked seeds of *Quercus mongolican*, *Pinus koraiensis*, and *Corylus mandshurica*) were placed on the ground approximately 30–80 cm in front of the camera. Ten days later, cameras were retrieved to collect and analyze the photos and videos.

### Recording and characterization of behaviors

Once the target animal was identified, the following observations were recorded, characterized, and later analyzed in order to construct the feeding and foraging behavior spectrum. The characterization included noting the type, frequency, and duration of behaviors exhibited during feeding and foraging. Due to the short duration of the video recording interval, 30 s, the video recording time, location of activity, and state of the target animal before and after the activity was used to assess whether the activity was continuous. The activity was considered to be continuous if the location and behavior did not change for the duration of the recording.

### Measuring of environmental variables: environment temperature, light intensity, sunrise, and sunset time

The environmental temperature was measured with the IR cameras, while light intensity was measured during early, middle, and late stage of the survey period, respectively. The measurements were taken once an hour only between 17:00 and 20:00 in the evening and 3:00 and 5:00 in the morning. For the sunrise and sunset time, we referred to local climate data and calculated the median.

### Statistical analysis

The data was tested for normality and equality of variance using the Kolmogorov-Smirnov and Levene’s test of homogeneity. Data was treated with respective tests depending on whether they met or did not met the assumptions of normality. The Kruskal-Wallis H test (nonparametric test) was used to compare the significant differences in behavior frequency, behavior duration, temperature, and light intensity among the three months. The t-test (Parametric test) or Mann-Whitney U test (nonparametric test) was used to test the differences between the different months. The association between feeding frequency, light intensity, and temperature was tested using the Pearson Correlation analysis. All data were expressed as mean ± sd and statistical significance was accepted when α < 0.05. All statistical analyses were conducted in SPSS 22.0 software.

## Results

### Species diversity and composition of Glires in the study area

Among the 6383 effective recorded activities in the video, 5618 were of *A. peninsulae*, accounting for 85.73% of all the Glire species in the area. Other recorded Glires include *Tamias sibiricus* with 523 records, squirrel with 226 records, and *Clethrionomys rufocanus* with 16, accounting for 7.98%, 3.45%, and 2.84%, respectively. Therefore, *A. peninsulae* was the absolute dominant species in the selected research plot.

### Classification and definition of foraging behaviors of *A. peninsulae*

*Apodemus peninsulae* behavior was analyzed from the video records. The main behavior patterns are as follows:

#### Motor behavior

A series of animal behaviors with obvious spatial displacements in different positions through various types of movement.

- Creep (slow movement). The abdomen of the body is on the ground, the forelimbs are stretched forward and flat on the ground, and then the two hindlimbs move forward simultaneously in a creeping movement. This kind of movement is the slowest, is used as the completion of short-distance movement and often occurs during foraging and feeding.
- Walk (medium-speed movement). The abdomen is off the ground; the limbs move alternately as the body stretches. The speed and distance in this kind of the movement are between creep and skip. It is used as complete short and medium-distance displacement and often occurs during foraging.
- Skip (fast movement). The hind limbs quickly kick off the ground, the body jumps forward and upward, and the displacement occurs in the form of a parabolic trajectory; the flying height is 10–30 cm, including a single skip and multiple consecutive skips, without other accompanying behaviors. This kind of movement often occurs during transport.

#### Feeding behavior

A series of behaviors exhibited by animals during feeding.

**Figure 1.**
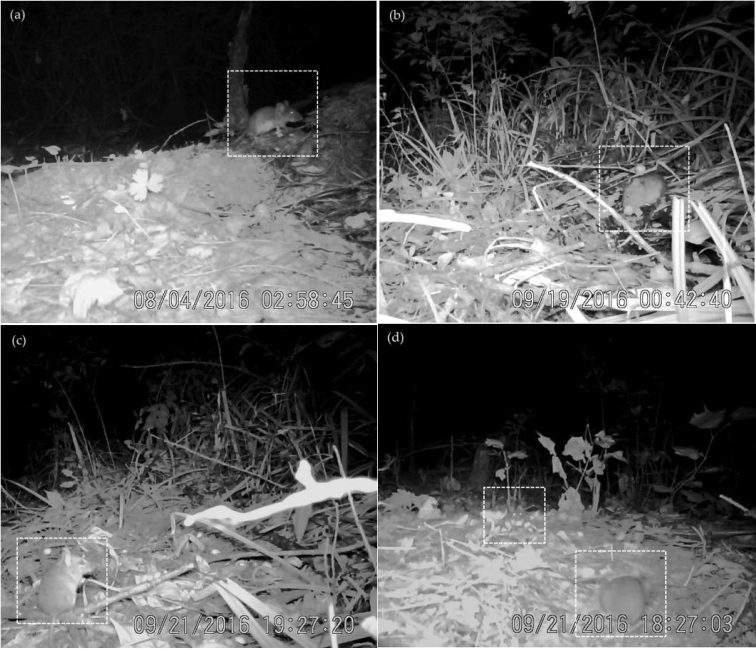
Some behaviors of *A. peninsulae.***a** forage **b** feeding **c** alert **d** coexistence.

- Forage: when animals are searching for food by walking and creeping using their senses of smell and vision. They usually search over a large area before finding a concentrated food source and continue to search in a smaller area after finding the food source. Animals often exhibit the following behaviors: sniffing, looking around, forearm digging, and other search actions with single short skips (Fig. 1a).
- Feeding: when animals are handling and eating food. When feeding, they usually do not move around, but they do occasionally creep, squat, or lie on the ground with hind limb support. The abdomen is on the ground, the back is raised, the forelimbs are on the ground or slightly raised and the two front paws grasp the food to assist in processing the seed coat and biting and chewing the food (Fig. 1b).
- Transport: when animals are moving their food from where it was found, but not eating or processing it immediately. Transport occurs after foraging, and animals usually leave quickly by running or skipping. The direction of transport is scattered.
- Hoarding: when animals, after transporting the food in a short distance, do not eat or process the food but stores it in a place. This can be a concentrated area (concentrated hoarding) or scattered spots (scattered hoarding) within the foraging area. Usually, the food is buried in the soil and the litter using the mouth to carry the food and forelimbs to dig.
- Clean: when animals self-groom by scratching to clean or groom the fur of the cheeks, neck, and chest with mouth, forelimbs, and hindlimbs. It usually occurs after or during feeding.

#### Sentinel behavior

A series of behaviors of animals exhibited in response to risks and disturbances in the environment, and vigilance in response to what is in the environment to avoid being depredated.

- Alert: animals immediately interrupt foraging, feeding and other activities; animals stay still by squatting while lifting the forelimbs, standing up slightly, bowing back, sniffing, listening, and observing the surrounding environment (Fig. 1c).
- Flee: animals immediately interrupt their ongoing activity and leave quickly by running fast with skip or continuous large distance skips after perceiving the danger or disturbance.
- Banishment: animals immediately interrupt ongoing activities when another rodent appears within the same area where it is foraging or feeding. Animals will quickly move to the other rodent by skipping to chase it away.
- Coexistence: when two rodents forage within the same area without any competition or showing any aggression towards each other. Both animals conduct their own forage or feeding behavior with at least 30 cm between one another (Fig. 1d).

### Feeding behavior strategy

#### Activity time of *A. peninsulae*

The start and end time of activity was consistent with sunrise and sunset; the percentage of activity time of *A. peninsulae* after sunset and sunrise was 100%, and 99.96%, respectively. Only two observations of activities that extended past sunrise by 30 minutes were found (Table 1). There was a seasonal, as well as night and day, effect on activity patterns, and much of the activity in summer was shorter than in autumn. From August to October, as the temperatures become cooler from August to October, the activity time starts to become longer. In August, *A. peninsulae* activity was at 37.1% of the whole day but increased to 52.3% and 52.2% in September and October, respectively.

**Table 1.** The activity time of *A. peninsulae* and partial climatic characteristics.

Month	Earliest time	Latest time	Sunset time	Sunrise time	Temperature (°C)	Illumination (Lx)
8	19:08:48	04:02:09	18:40	4:18	19.1 ± 2.2	371.4 ± 938.9
			(18:31–18:47)	(4:12–4:24)		
9	17:21:37	05:54:53	17:19	5:12	10.0 ± 1.6	64.6 ± 138.5
			(17:10–17:29)	(5:06–5:18)		
10	17:00:46	05:31:10	16:46	5:34	4.3 ± 1.4	16.3 ± 38.4
			(16:37–16:55)	(5:28–5:40)		

#### Feeding behavior frequency and duration

**Figure 2.**
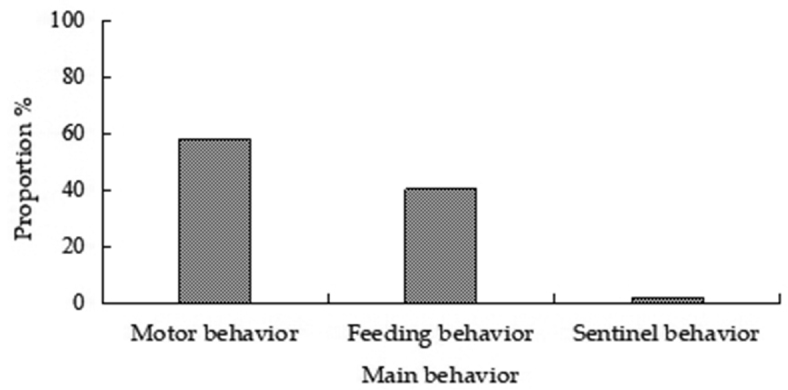
Proportion of three types behaviors of *A. peninsulae*.

**Figure 3.**
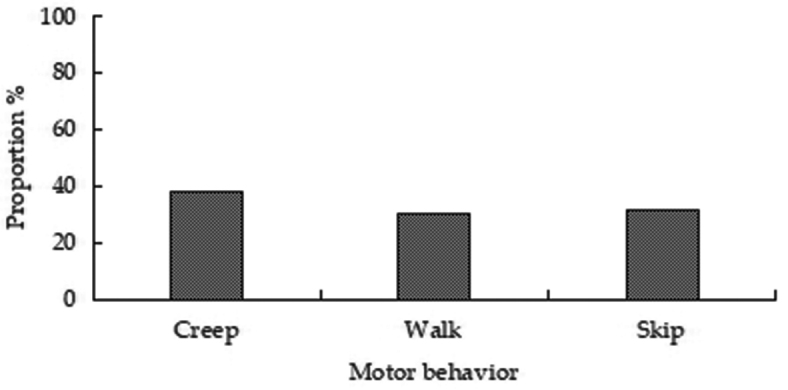
Proportion of motor behaviors of *A. peninsulae*.

**Figure 4.**
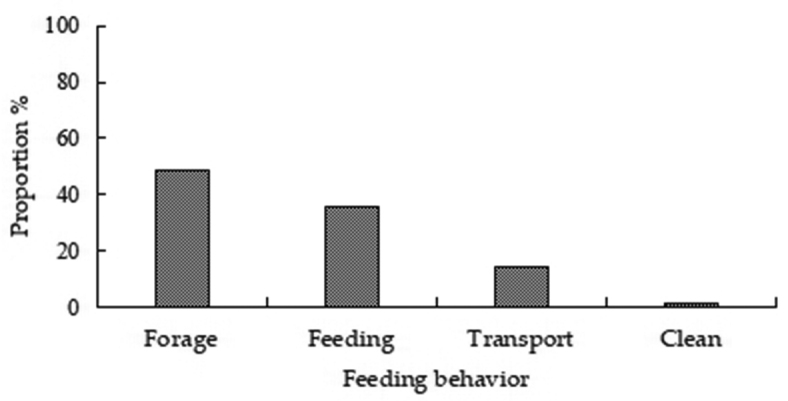
Proportion of feeding behaviors of *A. peninsulae*.

**Figure 5.**
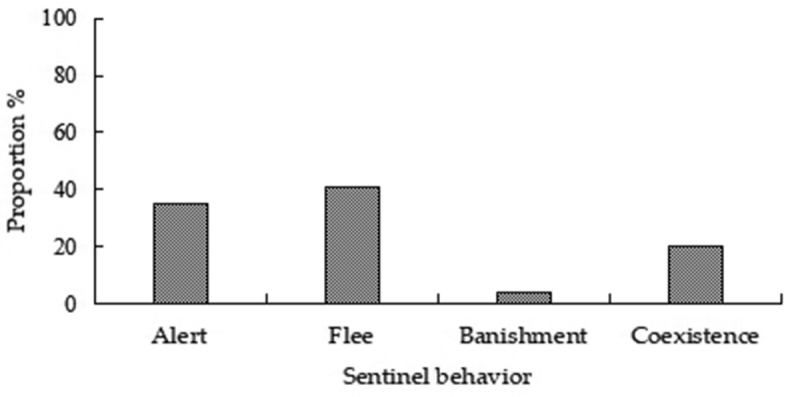
Proportion of sentinel behaviors of *A. peninsulae*.

Forage, feeding, and transport in feeding behavior were the main activities of *A. peninsulae* and were accompanied with motor and sentinel behavior. Of the total number of records (4403) of various types of behaviors, 57.96% (2552) were motor, 40.36% (1777) were feeding, and 1.68% (74) were sentinel behaviors (Fig. 2). Of motor behaviors, creep, walk, and skip accounted for 38.24%, 30.41%, and 31.35% of behavior, respectively (Fig. 3). Of feeding behaviors, foraging, feeding, transport, and cleaning accounted for 48.79%, 35.85%, 14.01%, and 1.35%, respectively (Fig. 4). Hoarding behavior after transport could not be recorded due to the limitation of camera monitoring range. In sentinel behavior, alert, flee, banishment, and coexistence accounted for 35.14%, 40.54%, 4.05%, and 20.27%, respectively (Fig. 5).

Feeding behavior frequency varied significantly in different months (*H* = 82.848, df = 2, *P* < 0.001). Total frequency was 21.6 ± 11.6 times/night (4.2–41.6 times / night, *N* = 26), of which 7.2 ± 2.8 times/night (4.2–10.5 times/night, *N* = 5) in August, 29.7 ± 7.8 times/night (17.9–41.6 times/night, *N* = 14) in September, which was the most frequent month, and 15.7 ± 7.5 times (4.5–25.4 times/night, *N* = 7) in October. The frequency of activities in August was significantly less than that in September (*t* = −9.220, *P* < 0.001) and October (*t* = −2.382, *P* < 0.05), and the frequency of activities in September was higher than October (*t* = 3.931, *P* < 0.01) (Table 2). Forage and feeding frequency were the highest in September, followed by October, and lowest in August (forage: *H* = 36.163, df = 2, *P* < 0.001; feeding: *H* = 10.262, df = 2, *P* < 0.01). The transport frequency in October was the highest, followed by September, and lowest in August (H = 6.018, df = 2, *P* < 0.05). There was no difference in the duration of forage in each month (*H* = 1.318, df = 2, *P* > 0.05), but the feeding duration was significant difference (H = 7.008, df = 2, *P* < 0.05) and the feeding duration in August was significantly longer than September and October (September: *Z* = −2.348, *P* < 0.05; October: *Z* = −2.602, *P* < 0.01).

**Table 2.** Frequency and duration of feeding behavior of *A. peninsulae* in different months.

Month	Frequency(time/night)				Duration (s)	
	Activity	Forage	Feeding	Transport	Forage	Feeding
8	7.2 ± 2.8	4.58 ± 2.87	4.17 ± 4.83	2.48 ± 1.86	42.78 ± 44.95	91.10 ± 118.02
9	29.7 ± 7.8	21.60 ± 10.02	12.30 ± 10.55	4.89 ± 5.90	47.05 ± 66.80	68.51 ± 102.98
10	15.7 ± 7.5	10.10 ± 8.36	11.35 ± 14.09	6.06 ± 4.83	29.16 ± 30.36	53.83 ± 88.72
Total	21.6 ± 11.6	10.84 ± 9.85	9.23 ± 11.17	4.37 ± 4.57	39.05 ± 51.63	63.58 ± 98.36

The frequency and duration of three feeding behaviors were all different. The average frequency of forage, feeding and transport was 10.84 ± 9.85 times/night, 9.23 ± 11.17 times/night, and 4.37 ± 4.57 times/night, respectively. The frequency of forage was significantly higher than feeding and transport (*H* = 23.092, df = 2, *P* < 0.001). Only in September did the frequency of three behaviors show significant differences from August to October (*H* = 25.614, df = 2, *P* < 0.001) (Table 2). The average duration of forage and feeding was 39.05 ± 51.63 s and 63.58 ± 98.36 s, respectively. Feeding duration was significantly longer than forage (*Z* = −6.704, *P* < 0.001) and it showed different differences in August, September, and October (*Z* = −3.930, *P* < 0.001; *Z* = −2.295, *P* < 0.05; *Z* = −5.478, *P* < 0.001).

#### Feeding activity rhythm

**Figure 6.**
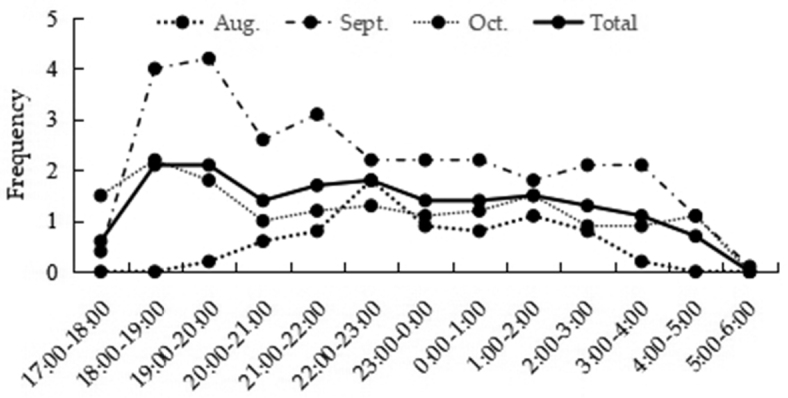
The night activity rhythm of *A. peninsulae* in different months.

Our observations show that the peak of feeding behavior occurs between 18:00 and 23:00 and varies from month to month. In August, only a single feeding peak was observed which started after 19:00, peaking between 22:00 and 23:00, and reducing after 3:00. In September and October, the activity started after 17:00, peaking between 18:00 and 20:00, which was earlier than that in August. In addition, the frequency of feeding was significantly higher than that in August, after 20:00, with a smooth curve and began to decrease after 4:00 (Fig. 6).

**Figure 7.**
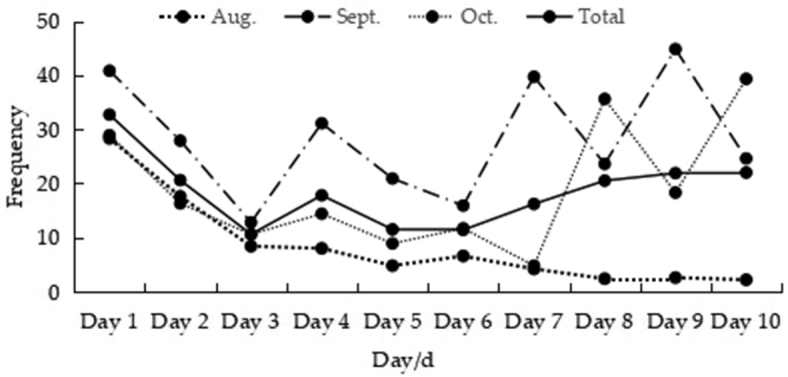
The activity rhythm of *A. peninsulae* during the study.

*A. peninsulae* showed a highest activity frequency on the first day it encountered a food source, with an average of 32.7 ± 7.1 times/night, then followed by daily feeding frequency between 10 and 22 times. This trend varied from month to month, with the feeding peak in August and decreasing thereafter. The feeding frequency showed the highest peaks on days 1, 4, 7, and 9 in September, and on days 1, 8, and 10 in October (Fig. 7).

#### Environmental factors and feeding behavior

**Figure 8.**
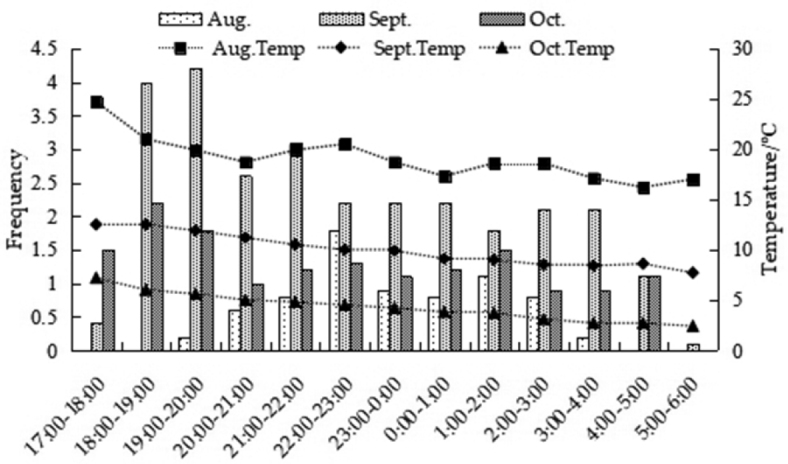
Interactions between temperature and feeding behavior of *A. peninsulae*.

**Figure 9.**
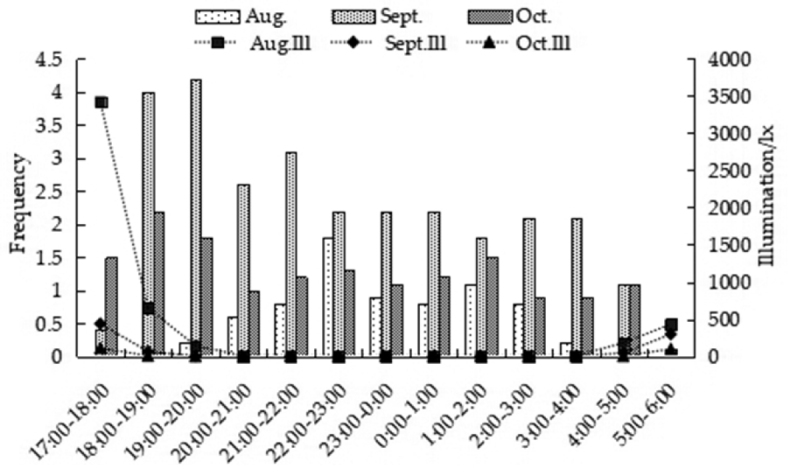
Interactions between illumination and feeding behavior of *A. peninsulae*.

From August to October, the temperature and light intensity decreased month by month. The temperature (*H* = 223.041, df = 2, *P* < 0.001) and light intensity (*H* = 14.812, df = 2, *P* < 0.001) from August to October showed significant differences. There was a strong positive association between temperature and feeding behavior during the month of September (*R* = 0.361, *P* < 0.001), but not during August and October (August: *R* = 0.118, *P* > 0.05; October: *R* = −0.036, *P* > 0.05; Fig. 8). Overall, feeding behavior was strongly associated with light intensity across the months, mainly September (*R* = 0.472, *P* < 0.001), and August (*R* = 0.294, *P* < 0.05), but this was not the case for October (*R* = 0.167, *P* > 0.05) (Fig. 9).

## Discussion

### Feeding behavior spectra of *A. peninsulae*

*Apodemus peninsulae* spends the majority of its active time feeding and exhibits some motor behaviors and sentinel behaviors during feeding. Our results show that foraging was the most frequent behavior and feeding was the longest behavior. *A. peninsulae* usually fed *in situ* when first encountering seeds, then transported seeds for later feeding or storage. The animals compensated for energy loss by reducing the frequency of foraging activity and spending more time to increase other activities such as sentinel behaviors so that they were more vigilant. Due to the limitation of the monitoring range of the camera, we were unable to follow post-transport feeding and hoarding activities, leading to an inaccurate calculation. We had similar results to Li et al. (2018) who showed that *A. peninsulae* was a scatter-hoarding animal which spent little time in feeding on seeds *in situ* (15.1%), but rather more time on post-transport feeding (20.4%) and hoarding (41.2%). Due to the existence of pressures, such as a complex and changeable environment, predation by natural enemies, and inter- and intraspecies competition, the combined feeding mode with *in situ* feeding, transport feeding, and scattered hoarding could help them to obtain greater benefits on the basis of reducing predation risk and decreasing competitive pressure (Clarke and Kramer 1994; Leaver and Daly 2001; Vander 2003, 2010; Vander and Beck 2012; Wang et al. 2013; Lichti et al. 2015).

Feeding and transport in *A. peninsulae* play two completely opposite roles in vegetation regeneration. On the one hand, feeding on a large number of seeds is harmful to forest regeneration (Lichti et al. 2015), while on the other, the transported and stored seeds exceed the demand, and any remaining seeds can potentially sprout and promote plant regeneration. In scatter-hoarding animals, it is common that caches are forgotten and plants can generate from those. Thus, the scatter-hoarding strategy effectively promotes plant regeneration and is a significant contributor to stabilizing plant population structure and maintaining the species diversity (Vander 2001; Preston and Jacobs 2009; Lichti et al. 2015). In response to the seasonal changes and the impacts on food resources, coupled with the north temperate climate, *A. peninsulae* showed different requirements to meet different life activities at different times. For example, in summer when resources were abundant and temperature and light intensity were suitable, *A. peninsulae* spent more time in feeding *in situ* and less time on other motor activities such as foraging and hoarding. However, when temperatures began to cool in the fall and resources were getting depleted, *A. peninsulae* began spending more time on foraging and transport activities, suggesting that they were storing food in order to successfully overwinter. This is an adaptation strategy in response to the seasonal changes of food and environment and is important for survival and reproduction of the species now and in the future (Vander 2001; Murray et al. 2006; Chang et al. 2010; Lichti et al. 2015).

### Activity rhythm of *A. peninsulae* and affecting factors

Activity rhythm is a comprehensive adaptation to obtain the greatest survival benefits under various conditions and is affected by a variety of internal and external factors. Among them, solar radiation, light intensity, and environmental temperature are the main factors that affect animal activity rhythm (Roll et al. 2006). The change of the length of day and night is an effective constraining factor of the distribution of an animal’s activity time; thus, it is particularly important to allocate its activity time appropriately to the available time (Dunbar 1992). Studies have confirmed that light was an important factor for the activities of Glires, and strictly nocturnal animals were inactive during the daylight period. Our study showed that the frequency of feeding behavior of *A. peninsulae* was strongly correlated with light intensity, showing an increased frequency of activity with a reduction in light intensity. This indicates that the nocturnal activity of these rodents is influenced by the intensity of light, since they are adapted to functioning at low light, twilight, and dusk. Most *Apodemus* spp. are either nocturnal, crepuscular, or both (Wolton 1983). Thus, it is unsurprising that these behaviors are consistent with what we observed in our study. In addition, the activity time of *A. peninsulae* showed seasonal differences, with shorter activity time in summer and longer time in autumn. The difference between the two seasons was up to 4 h, supporting the idea that temperature also influences activity patterns (Park et al. 2014).

Temperature is known to have considerable effect on changes in the daily activity rhythm in animals (Watanuki and Nakayama 1993; Corp et al. 1997; Hu et al. 2002). Environment temperature has an important effect on the night activity of Glires (Corp et al. 1997), with rodent species adjusting their foraging times and activities in response to changes in temperature (Hu et al. 2002). Usually, *A. peninsulae* is more active at relatively warm temperatures (the average daily temperature is 0 °C) (Park et al. 2014), but it can adapt well to changes in environmental temperatures without having severe impact on its activities. As *A. peninsulae* does not hibernate in the cold months, it likely survives by greatly reducing foraging and feeding activities (Chang et al. 2010; Park et al. 2014).

### Benefits and applications of IR cameras in the ecological studies

Studies under laboratory conditions have shown that *A. peninsulae* can be active both day and night in the spring (May), and the activity duration at night is more than that in day (Ji et al. 2005) , suggesting that seasonal difference may have an effect on animal activity rhythm. However, our data captured by IR cameras or radio monitoring with little human interference, shows more realistic behavioral patterns than laboratory studies. IR cameras reveal the strictly nocturnal and crepuscular lifestyle of *A. peninsulae*, and while it is relatively difficult to observe the behavior of nocturnal animals in the natural environment, IR cameras can provide an effective method to capture realistic information.

IR cameras have been used to assess species diversity survey and population density estimation in large mammals (Rowcliffe et al. 2008; Mondol et al. 2009; Zhang et al. 2013). However, IR cameras are less used in studies on small rodents (Zhang et al. 2013). Nocturnal small mammals are difficult to identify to individuals through photos or videos taken at night due to the lack of “natural marks”. Identifying gender and age is just as difficult (Zhang et al. 2013). IR cameras have their own shortcomings. The effectiveness of these cameras depend on the probability, for example, that the study animal will appear at the site, the activities will be within the field of vision of the camera, and that the photos and videos will be in focus and clear; the cameras will also capture a high number of repeat records of the same individuals (Mondol et al. 2009; O’Connell et al. 2010; Zhang et al. 2013). Here, we found that the behaviors recorded by IR camera were fewer in number than in laboratory studies, but we captured more information on abundance compared to traditional surveys in natural environment (O’Connell et al. 2010; Liu et al. 2015; Li et al. 2018; Claridge et al. 2019; Nichols et al. 2019).

## Conclusion

Infra-red cameras were used to record in natural conditions the feeding behavior of a small species of Glires, *A. peninsulae*, with both nocturnal and crepuscular behavior. In the behavior spectra, feeding behavior followed by motor and sentinel behaviors were the main activities of this species. It spent the majority of its active time feeding and foraging. The behavior was influenced by light intensity, suggesting a preference for crepuscular periods of the day. This species’ activities had significant seasonal differences and is seen as an adaptation strategy in response to seasonal changes in food and the environment. Our results show that IR cameras are highly useful in ecological studies of species of Glires that are not easily observable. IR cameras are able to capture much valuable information on ecological functions.
